# Assessing health-related quality of life in patients with interstitial lung diseases with and without lung transplantation using the GR-Scale

**DOI:** 10.1186/s12890-025-04101-1

**Published:** 2026-01-10

**Authors:** Sina Stoltefuß, Gabriela Leuschner, Tobias Veit, Jeremias Götschke, Katrin Milger, Teresa Kauke, Alexandra Lenoir, Nikolaus Kneidinger, Jürgen Behr

**Affiliations:** 1 Department of Medicine V, LMU University Hospital, Comprehensive Pneumology Center, LMU Munich, Member of the German Center for Lung Research (DZL), Marchioninistr. 15, 81337 Munich, Germany; 2 Department of Thoracic Surgery, LMU University Hospital, LMU Munich, 81337 Munich, Germany; 3 Division of Respiratory Medicine, Department of Internal Medicine, Lung Research Cluster, Medical University of Graz, 8036 Graz, Austria

**Keywords:** Interstitial lung disease, ILD, Lung transplantation, Graft rejection, CLAD, Health related quality of life, HRQoL, Patient reported outcome measures, PROM

## Abstract

**Introduction:**

Lung transplantation (LTx) aims to improve the health-related quality of life (HRQoL). We applied the GR-Scale for the first time in patients after LTx due to interstitial lung diseases (ILDs), compared it longitudinally with non-transplanted patients, and evaluated the impact of chronic allograft dysfunction (CLAD) on the HRQoL.

**Methods:**

In this prospective longitudinal cohort pilot study, we compared HRQoL by utilizing the GR-Scale between non-transplanted patients (*n* = 32) and transplanted patients (*n* = 26). In a second step, we compared HRQoL between transplanted patients with (*n* = 11) and without CLAD (*n* = 42). A follow-up assessment was conducted after 3 to 6 months.

**Results:**

Statistically significant differences in HRQoL were observed between patients after LTx and non-transplanted patients, with worse HRQoL in non-transplanted patients, at both baseline (*p* < 0.001) and follow up (*p* < 0.001). Furthermore, the GR-Scale found statistically significant worse HRQoL in transplanted patients with CLAD compared to those without, both at baseline (*p* = 0.014) and follow-up (*p* = 0.011). Additionally, significant differences were found in the individual items of the GR-Scale, with physical symptoms showing a more pronounced difference than psychological symptoms in both analyses. No significant differences were observed between the groups regarding age and sex.

**Conclusion:**

Our pilot study suggests that the GR-Scale is an easy-to-use and valuable tool to assess HRQoL in lung transplant recipients providing important additional information for clinical evaluation of this patient population, also longitudinally.

**Trial registration:**

Our study was retrospectively registered in the German Clinical Trial Register (DRKS) on 02.11.2022 (DRKS-ID: DRKS00030599).

## Introduction

Interstitial lung diseases (ILDs) often lead to a limited health-related quality of life (HRQoL) as they cause a major burden for patients and affect their lives in many ways [1–4]. Usually, ILD symptoms begin insidiously and increase over time. The most common symptoms include shortness of breath, cough, and fatigue [5, 6]. Particularly as the disease progresses, the HRQoL is increasingly negatively affected. This is caused by the symptoms of the disease, but also factors such as therapy-related aspects, reduced social participation, dependence on other people, the prognosis and mental health restrictions play an important role [1].

The respective therapy of ILD varies depending on the specific type of disease and its severity [5, 7]. In selected patients with very severe and advanced disease, lung transplantation (LTx) is an important and the only potentially curative treatment option [5, 8]. The main goals of LTx are to prolong patients’ lives and improve their quality of life [9–13]. Prior studies have shown a significant improvement in HRQoL at 3 to 6 months after LTx [13, 14].

Over time, however, the improved HRQoL may decline again due to various causes, such as medication-related side effects, comorbidities, infection, and depression. Another important factor is the occurrence of chronic lung allograft dysfunction (CLAD) [13, 14]. CLAD is often regarded as chronic rejection which is defined by a substantial and persistent decline (> 20%) in measured forced expiratory volume in one second (FEV1) from the reference (best-FEV1 after LTx) value. The staging of CLAD (into stages 0 to 4) is also based on the FEV1 value [15, 16]. The probability of the occurrence of CLAD increases with time after LTx. After 2 years 22%, after 5 years 50%, and after 10 years 77% of lung transplant recipients are affected [17, 18]. CLAD has two main clinical presentations, bronchiolitis obliterans syndrome (BOS) and restrictive allograft syndrome (RAS), which can overlap, leading to mixed or undefined phenotypes. The most common phenotype is BOS (with up to 80%), while RAS accounts for approximately 30% and the phenotypes mixed and undefined are rather rare [15, 16, 19, 20]. Symptoms include cough, shortness of breath, and fatigue, with RAS progressing more rapidly and severely than BOS [21]. Treatment options are limited, and median survival is around 2.5 years, with RAS having a poorer prognosis than BOS [15, 16, 19, 20].

HRQoL has become increasingly relevant as a patient-reported outcome and endpoint in clinical trials for both non-transplanted ILD patients and those after LTx [14, 22]. It can be assessed using patient-reported outcome measures (PROMs), usually questionnaires, which capture the patient’s perspective on their symptoms and daily life with the disease, without the interpretation by another person. Compared to other routine measures, such as lung function parameters or chest imaging, PROMs can capture how patients “feel and function” in their lives, offering a more complete understanding of the disease’s impact. These other routine measures are clearly important but may not fully capture the patient experience and can differ from patient’s perceptions [2, 13, 23, 24]. This underlines the fact that relying solely on these objective measurements can lead to misinterpretations and emphasises the importance of PROMs and the assessment of HRQoL.

Due to length and complexity of existing questionnaires, Scallan et al. designed the Raghu scale for pulmonary fibrosis (R-Scale-PF), which is originally intended to assess the HRQoL in patients with idiopathic pulmonary fibrosis (IPF). This simple numerical rating scale assesses the severity of five symptoms (cough, shortness of breath, fatigue, depressed mood, overall sense of wellbeing) in the last two weeks. Scallan et al. have evaluated the questionnaire in 100 patients with IPF and comparable results to the established questionnaires (K-BILD) questionnaire and the EuroQol Five-Dimensional Five-Level questionnaire (EQ-5D-5 L) where found [25]. In a previous study we have developed a German version of the R-Scale-PF (GR-Scale) and have evaluated its validity in various ILD entities [26].

The aim of our prospective longitudinal cohort pilot study was to apply the GR-Scale for the first time in patients who had undergone LTx due to ILD. We used the GR-Scale to compare the HRQoL between patients after LTx and non-transplanted patients with ILDs, and subsequently assessed the impact of the occurrence of CLAD on the HRQoL in patients after LTx.

## Methods

### Study design and population

The study populations were recruited at the LMU University Hospital Munich, Germany. We included consecutive patients, age 18 years or older, with a consensus diagnosis of ILD, including IPF, chronic hypersensitivity pneumonitis (cHP), non-specific interstitial pneumonia (NSIP) and unclassifiable interstitial lung disease (uILD) and those who have received double-LTx due to these diagnoses. All diagnoses were made in accordance with current international criteria [27]. Patients with an acute exacerbation of their underlying ILD or any other acute medical event at the time of assessment were excluded. Our study comprises two separate analyses to assess the impact of LTx on the HRQoL in patients with ILDs based on four independent patient cohorts: the first analysis compared the HRQoL between non-transplanted and transplanted patients with ILDs, while the second compared the HRQoL between transplanted patients with and without CLAD. To assess the HRQoL, we used the GR-Scale, as previously published [25, 26]. In brief, the GR-Scale is a questionnaire consisting of 5 items (cough, shortness of breath, fatigue, depressed mood, overall sense of wellbeing), with scores range from 0 to 10 for each item and from 0 to 50 for the total score, where higher scores indicate greater limitations [25, 26]. The patients were interviewed twice with the GR-Scale. The follow-up interview took place after 3 to 6 months, consistent with guideline recommended intervals for ILD patients and the routine follow-up practice at our hospital, and was included to capture potential changes in HRQoL and to evaluate the consistency across repeated measurements. All patients completed the questionnaire as self-assessment, without the help of health care professionals. Pulmonary function data and comorbidities were reported to characterize the study population but were not part of the analyses. The comorbidities were systematically recorded from the patients’ medical records. All clinically documented diagnoses were considered.

#### Comparison of HRQoL in ILD patients with and without lung transplantation

For this comparison, we included consecutive patients with the above-mentioned diagnoses and those who received double-LTx due to these ILD diagnoses. Transplanted patients were included in the study if the transplantation had been performed more than 3 months but no longer than 3 years prior to the baseline examination. We used this time frame assuming that the benefit from LTx is maximized in this period, as acute problems have been solved and long-term sequalae are not yet dominant. In order to substantiate this assumption and identify any potential influence of the chosen time frame, we conducted a correlation analysis between time since transplantation and the GR-Scale total scores, at baseline and follow-up. For non-transplanted patients, status on the lung transplant waiting list was not considered an inclusion or exclusion criterion, as the study focused on the clinical comparison between transplanted and non-transplanted ILD patients and not on pre-transplant evaluation characteristics. To identify potential confounders, we analyzed group differences in terms of age and sex. We then compared the GR-Scale results of the two groups with each other. Both at baseline and at follow-up, we compared the 5 individual items and the total score.

#### Comparison of HRQoL in lung transplant patients with and without CLAD

For this analysis, we included consecutive patients who had undergone double-LTx due to one of the above-mentioned diagnoses. The time since transplantation was not an inclusion or exclusion criterion for this analysis. The included transplanted patients were divided into two groups based on the presence or absence of CLAD, which is defined by a substantial and persistent decline (> 20%) in measured FEV1 from the reference (best-FEV1 after LTx) value and exclusion of other causes [15]. Again, we analyzed differences between the groups in terms of age and sex, to identify potential confounders and we then compared the GR-Scale results (5 items and total score) at baseline and at follow-up.

### Statistical analysis

Descriptive statistics were calculated for all groups and for both baseline and follow-up variables, using mean and standard deviation to describe continuous variables and absolute frequencies and percentages for categorical variables.

To compare variables between two groups, we chose the independent two-sample t-test for continuous variables, reporting the effect size as Cohens’ d. These effect sizes (Cohen’s d) were calculated using the point estimate, based on the pooled standard deviation of the two compared groups and interpreted following Cohen’s guidelines with 0.2 = small, 0.5 = medium and ≥ 0.8 = large [28]. For categorical variables, we applied the chi-square-test, reporting the effect size as Cramér’s V.

To analyze potential correlations, we used the Pearson correlation coefficient (r) for continuous variables and reported both r-values and corresponding *p*-values.

For statistical analysis IMB SPSS statistics, version 29 (IBM, Armonk, NY, USA) was used, with *p* < 0.05 considered statistically significant.

### Ethical approval

This study was approved by the ethics committee of the medical faculty of LMU Munich (project number 22–0651). Written informed consent was obtained from all patients prior to enrolment.

## Results

### HRQoL in ILD patients with and without lung transplantation

#### Baseline characteristics

Between October 2022 and December 2022, the GR-Scale was completed by 32 patients with a multidisciplinary discussion (MDD) based diagnosis of ILD (group 1) and by 26 patients who had previously received a LTx due to a MDD-based diagnosis of ILD (group 2), all of whom presented for routine clinical check at our tertiary hospital. All patients completed the GR-Scale questionnaire without missing information.

The patients’ demographics and baseline characteristics are shown in Table 1. In group 1, 27 (84.38%) patients completed the questionnaire a second time after 4.36 ± 1.21 months. In group 2, 26 (100%) patients completed the questionnaire again after 4.41 ± 1.14 months. The GR-Scale results of the two groups at follow-up are also shown in Table 1.

**Table 1:** Baseline characteristics and GR-Scale results of groups 1 and 2

Characteristics	Group 1 (non-transplanted ILD)	Group 2 (transplanted)	*p*-value
Patients n	32	26	
Male: female n	19:13	20:6	0.16
Age, year	65.44 ± 11.14	61.23 ± 6.48	0.09
Time since LTx (years)	-	1.31 ± 0.72	
ILD subtype n (%)			
cHP	10 (31.25)	7 (26.92)	0.719
IPF	9 (28.13)	11 (42.31)	0.258
NSIP	8 (25.00)	5 (19.23)	0.6
uILD	5 (15.62)	3 (11.54)	0.654
ILD subtype distribution			0.726
Comorbidities	3.28 ± 2.2	5.04 ± 2.18	0.004
Lung function at baseline			
TLC, % predicted	66.02 ± 18.02	75.23 ± 16.06	0.059
FVC, % predicted	70.56 ± 18.5	79.16 ± 19.21	0.093
FEV1, % predicted	72.28 ± 20.82	81.89 ± 22.78	0.103
Lung function at follow-up			
TLC, % predicted	67.62 ± 18.1	76.19 ± 16.31	0.076
FVC, % predicted	69.62 ± 20.6	80.04 ± 19.53	0.065
FEV1, % predicted	71.96 ± 21.94	81.87 ± 22.34	0.11
GR-Scale results at baseline			
Cough	3.69 ± 2.89	1.71 ± 1.84	0.004
Shortness of breath	5.34 ± 2.66	2.69 ± 1.91	< 0.001
Fatigue	4.48 ± 2.49	2.73 ± 1.96	0.005
Depressed mood	2.44 ± 2.35	1.44 ± 2	0.092
Overall sense of wellbeing	4.3 ± 2.31	3 ± 2.01	0.028
Total Score	20.25 ± 9.2	11.58 ± 7.12	< 0.001
GR-Scale results at follow-up			
Cough	3.69 ± 2.77	2.08 ± 1.83	0.016
Shortness of breath	5.35 ± 2.7	2.77 ± 2.46	< 0.001
Fatigue	4.65 ± 2.83	2.75 ± 2.21	0.009
Depressed mood	2.98 ± 2.45	1.33 ± 1.86	0.008
Overall sense of wellbeing	4.2 ± 2.1	2.52 ± 1.83	0.003
Total score	20.87 ± 10.56	11.44 ± 7.97	< 0.001

Data are presented as mean ± SD, unless otherwise stated

*Definition of abbreviations* *ILD*  interstitial lung disease, *cHP*  chronic hypersensitivity pneumonitis, *IPF*  idiopathic pulmonary fibrosis, *NSIP*  nonspecific interstitial pneumonitis, *uILD*  unclassifiable interstitial lung disease, *TLC*  total lung capacity, *FVC*  forced vital capacity, *FEV1*  forced expiratory volume in one second

Group 1 = non-transplanted patients with ILD, group 2 = transplanted patients due to ILD

#### Comparison of age and sex

The independent two-sample t-test showed no significant difference between the two groups in terms of age (*p* = 0.09). The mean age in group 1 was 65.44 ± 11.14 years and in group 2 61.23 ± 6.48 years. The mean difference was 4.21 years (95% CI [-0.74, 9.15]) and the effect size, Cohen`s d, was 0.45.

Also for sex, no significant difference between the groups could be determined using the chi-square-test (χ²(1) = 2.01, *p* = 0.16). In group 1 40.63% were females and in group 2 23.08% were females. The effect size, Cramér’s V, was 0.19.

These results indicate comparability between the groups regarding age and sex distribution.

#### Comparison of the GR-Scale results at baseline and follow-up

At baseline, significant differences were found for the items “cough”, “shortness of breath”, “fatigue” and “overall sense of wellbeing”, as well as for the total score between the two groups. All these mentioned values were statistically significant higher in group 1, indicating a higher burden of disease. Only the item “depressed mood” showed no statistically significant differences between the two groups. However, the value was numerically higher in group 1.

At follow-up, differences were found for all 5 items (cough, shortness of breath, fatigue, depressed mood and overall sense of wellbeing) as well as in the total scores. All values were statistically significant higher in group 1.

No statistically significant correlation was found between time since transplantation and the GR-Scale total score, neither at baseline (*r* = 0.275, *p* = 0.174) nor at follow-up (*r* = 0.096, *p* = 0.642). Indicating that the time since transplantation has not significantly influenced the GR-Scale results of the transplanted patients (Table 2).

**Table 2:** Comparison of the GR-Scale results at baseline and follow-up between groups 1 (non-transplanted ILD) and 2 (transplanted)

	Group	Mean ± SD GR-Scale total score	Mean difference (95% CI)	*p*-value	ES
GR-Scale results at baseline					
Cough	1	3.69 ± 2.89	1.98 (0.66–3.29)	0.004	0.8
	2	1.71 ± 1.85			
Shortness of breath	1	5.34 ± 2.66	2.65 (1.4–3.9)	< 0.001	1.13
	2	2.69 ± 1.91			
Fatigue	1	4.48 ± 2.49	1.75 (0.55–2.95)	0.005	0.77
	2	2.73 ± 1.96			
Depressed mood	1	2.44 ± 2.35	1.0 (-0.17-2.16)	0.092	0.45
	2	1.44 ± 2.0			
Overall sense of wellbeing	1	4.3 ± 2.31	1.3 (0.14–2.45)	0.028	0.6
	2	3.0 ± 2.01			
Total score	1	20.25 ± 9.2	8.67 (4.26–13.08)	< 0.001	1.04
	2	11.58 ± 7.12			
GR-Scale results at follow-up					
Cough	1	3.69 ± 2.77	1.61 (0.31–2.91)	0.016	0.68
	2	2.08 ± 1.83			
Shortness of breath	1	5.35 ± 2.7	2.58 (1.16–4.01)	< 0.001	1.0
	2	2.77 ± 2.46			
Fatigue	1	4.65 ± 2.83	1.9 (0.49–3.3)	0.009	0.75
	2	2.75 ± 2.23			
Depressed mood	1	2.98 ± 2.45	1.65 (0.45–2.86)	0.008	0.76
	2	1.33 ± 1.86			
Overall sense of wellbeing	1	4.2 ± 2.1	1.68 (0.6–2.77)	0.003	0.85
	2	2.52 ± 1.83			
Total score	1	20.87 ± 10.56	9.43 (4.25–14.6)	< 0.001	1.0
	2	11.44 ± 7.97			

*Definition of abbreviations SD* = Standard deviation, *CI*  Confidence interval, *ES*  effect size (Cohen’s d)

Group 1 = non-transplanted patients with ILD, group 2 = transplanted patients due to ILD

### HRQoL in lung transplant patients with and without CLAD

#### Baseline characteristics

Between October 2022 and December 2022, the GR-Scale was completed by 42 patients who had undergone LTx due to a MDD based diagnosis of ILD without CLAD (group 3) and by 11 patients with CLAD (group 4). All patients have undergone routine clinical care at our tertiary hospital and all of them completed the GR-Scale without missing information.

The patients’ demographics and baseline characteristics are shown in Table 3. In group 3, 41 (97.62%) patients completed the questionnaire a second time after 4.57 ± 1.08 months. In group 4, 11 (100%) patients completed the questionnaire again after 4.9 ± 0.8 months. The GR-Scale results of the two groups at follow-up are also shown in Table 3.

**Table 3:** Baseline characteristics and GR-Scale results of groups 3 and 4

Characteristics	Group 3 (no CLAD)	Group 4 (CLAD)	*p*-values
Patients n	42	11	
Male: female n	32:10	6:5	0.16
Age, year	60.52 ± 8.25	60.09 ± 10.65	0.885
Time since LTx (years)	4.0 ± 3.73	8.34 ± 6.41	0.005
ILD subtype n (%)			
cHP	14 (33.33)	3 (27.27)	0.701
IPF	18 (42.86)	6 (54.55)	0.488
NSIP	6 (14.29)	2 (18.18)	0.748
uILD	4 (9.52)	0 (0)	0.287
ILD subtype distribution			0.683
Comorbidities	4.9 ± 2.0	4.91 ± 2.95	0.995
Lung function at baseline			
TLC, % predicted	74.73 ± 19.1	68.53 ± 8.11	0.35
FVC, % predicted	84.71 ± 23.19	70.42 ± 17.19	0.062
FEV1, % predicted	84.93 ± 25.03	65.95 ± 16.63	0.022
Lung function at follow-up			
TLC, % predicted	78.16 ± 18.1	64.67 ± 11.47	0.038
FVC, % predicted	83.65 ± 22.11	66.03 ± 14.82	0.027
FEV1, % predicted	82.96 ± 24.71	63.79 ± 17.66	0.032
CLAD phenotype n (%)			
BOS	-	8 (72.73)	
RAS	-	1 (9.09)	
Mixed	-	1 (9.09)	
Undefined	-	1 (9.09)	
CLAD stage at baseline n (%)			
1	-	9 (81.82)	
2	-	2 (18.18)	
CLAD stage at follow up n (%)			
1	-	7 (63.64)	
2	-	4 (36.36)	
GR-Scale results at baseline			
Cough	1.6 ± 1.66	1.36 ± 1.45	0.676
Shortness of breath	2.57 ± 2.26	4.32 ± 3.04	0.039
Fatigue	2.44 ± 2.1	5.09 ± 2.52	< 0.001
Depressed mood	1.3 ± 1.79	2.14 ± 1.87	0.182
Overall sense of wellbeing	2.92 ± 2.12	4.18 ± 1.93	0.079
Total Score	10.83 ± 7.52	17.09 ± 6.18	0.014
GR-Scale results at follow-up			
Cough	1.9 ± 1.74	2.27 ± 2.68	0.58
Shortness of breath	2.43 ± 2.36	4.96 ± 3.09	0.005
Fatigue	2.59 ± 2.3	5.14 ± 2.78	0.003
Depressed mood	1.37 ± 1.68	1.5 ± 1.47	0.811
Overall sense of wellbeing	2.61 ± 1.97	4.23 ± 2.16	0.022
Total score	10.89 ± 8.13	18.09 ± 7.75	0.011

Data are presented as mean ± SD, unless otherwise stated

*Definition of abbreviations* *LTx*  lung transplantation,* ILD*  interstitial lung disease,* cHP*  chronic hypersensitivity pneumonitis, *IPF*  idiopathic pulmonary fibrosis, *NSIP*  nonspecific interstitial pneumonitis, *uILD*  unclassifiable interstitial lung disease, *TLC*  total lung capacity,* FVC*  forced vital capacity, *FEV1*  forced expiratory volume in one second, *CLAD*  chronic lung allograft dysfunction, *BOS*  bronchiolitis obliterans syndrome, *RAS*  restrictive allograft syndrome

Group 3 = transplanted patients without CLAD, group 4 = transplanted patients with CLAD

#### Comparison of age and sex

The independent two-sample t-test showed no significant difference between the two groups in terms of age (*p* = 0.885). The mean age in group 3 was 60.52 ± 8.25 years and in group 4 60.09 ± 10.65 years. The mean difference was 0.433 years (95% CI [-5.53, 6.4]) and the effect size, Cohen`s d, was 0.05.

Also for sex, no significant difference between the groups could be determined using the chi-square-test (χ²(1) = 2.01, *p* = 0.16). In group 3 23.81% were females and in group 4 45.5% were females. The effect size, Cramér’s V, was 0.19.

These results indicate comparability between the groups regarding age and sex distribution.

#### Comparison of the GR-Scale results at baseline and follow-up

At baseline, statistically significant differences were found for the items “shortness of breath” and “fatigue”, as well as for the total score. All these values were higher in group 4. The items “cough”, “depressed mood” and “overall sense of wellbeing” showed no statistically significant difference between the two groups. However, the values for the items “depressed mood” and “overall sense of wellbeing” were numerically higher in group 4. Only the value for the item “cough” was numerically higher in group 3.

At follow-up, statistically significant differences were found for the items “shortness of breath”, “fatigue” and “overall sense of wellbeing”, as well as for the total score. The values were higher in group 4. The items “cough” and “depressed mood” showed no statistically significant difference between the groups. Both values were numerically higher in group 4 (Table 4). 

**Table 4:** Comparison of the GR-Scale results at baseline and follow-up between groups 3 (no CLAD) and 4 (CLAD)

	Group	Mean ± SD GR-Scale total score	Mean difference (95% CI)	*p*-value	ES
GR-Scale results at baseline					
Cough	3	1.6 ± 1.66	0.23 (-0.87-1.34)	0.676	0.14
	4	1.36 ± 1.45			
Shortness of breath	3	2.57 ± 2.26	-1.75 (-3.4-(-0.09))	0.039	0.72
	4	4.32 ± 3.04			
Fatigue	3	2.44 ± 2.1	-2.65 (-4.14-(-1.16))	< 0.001	1.21
	4	5.09 ± 2.52			
Depressed mood	3	1.3 ± 1.79	-0.83 (-2.05-0.4)	0.182	0.46
	4	2.14 ± 1.87			
Overall sense of wellbeing	3	2.92 ± 2.12	-1.27 (-2.68-0.15)	0.079	0.61
	4	4.18 ± 1.93			
Total score	3	10.83 ± 7.52	-6.26 (-11.2-(-1.31))	0.014	0.86
	4	17.09 ± 6.18			
GR-Scale results at follow-up					
Cough	3	1.9 ± 1.74	-0.37 (-1.7-0.97)	0.58	0.18
	4	2.27 ± 2.68			
Shortness of breath	3	2.43 ± 2.36	-2.53 (-4.25-(-0.81))	0.005	1.0
	4	4.96 ± 3.09			
Fatigue	3	2.59 ± 2.3	-2.55 (-4.19-(-0.91))	0.003	1.06
	4	5.14 ± 2.78			
Depressed mood	3	1.37 ± 1.68	-0.13 (-1.25-0.98)	0.811	0.08
	4	1.5 ± 1.47			
Overall sense of wellbeing	3	2.61 ± 1.97	-1.62 (-2.99-(-0.25))	0.022	0.81
	4	4.23 ± 2.16			
Total score	3	10.89 ± 8.13	-7.2 (-12.69-(-1.71))	0.011	0.89
	4	18.09 ± 7.75			

*Definition of abbreviations* *SD*  Standard deviation, *CI*  Confidence interval, *ES*  effect size (Cohen’s d)

Group 3 = transplanted patients without CLAD, group 4 = transplanted patients with CLAD

## Discussion

In our pilot study we applied the GR-Scale for the first time in patients who had undergone LTx due to ILD. We used the GR-Scale in age and sex comparable cohorts to investigate the HRQoL in ILD patients after LTx and non-transplanted ILD patients. We also used the GR-Scale to assess the impact of the occurrence of CLAD on the HRQoL in patients after LTx.

The GR-Scale successfully assessed expected differences in the HRQoL between non-transplanted patients and patients after LTx, both at baseline and at follow-up, whereby the non-transplanted patients showed higher values in the total score and thus a more severely impaired HRQoL. Statistically significant differences were also found in the individual items, except for the item “depressed mood” at baseline. The group of non-transplant patients also showed higher values for the individual items, indicating a greater burden of symptoms. Furthermore, the GR-Scale was also able to assess the expected difference in HRQoL between transplanted patients with and without CLAD, with statistically significant differences in the total scores as well as in “shortness of breath”, “fatigue” and, at follow-up, “overall sense of wellbeing”. Patients with CLAD had higher values in the overall score and the individual items and thus a more impaired HRQoL and higher individual symptom burden.

Our results comparing patients with and without LTx (group 1 and 2) are in line with the findings of previous studies, in which an improvement in HRQoL was also observed after LTx, although different methodologies were used [13, 14]. Other studies have also shown that, in particular, physical symptoms tend to improve more after LTx [29, 30]. Since the GR-Scale reflected these differences both at baseline and at follow-up, this indicates that the GR-Scale can consistently capture the improvement in HRQoL across repeated measurements.

The only item without a statistically significant difference at baseline was “depressed mood”. An explanation for this could be the existing psychosocial burden after LTx, including constant need for medical monitoring, the fear of rejection and repeated hospitalizations, which in turn worsens HRQoL [14]. Our baseline results are consistent with previous studies, which found no statistically significant improvement in psychological symptoms compared to physical ones after LTx [29, 30]. However, some studies do report improvement across all domains, including psychological symptoms [31]. In summary, differences in psychological symptoms between non-transplanted and transplanted patients tend to be less pronounced than physical items and overall HRQoL. More sophisticated psychological questionnaires are probably needed to address this difference in more detail.

Various factors, especially the occurrence of CLAD with increasing symptoms like shortness of breath, fatigue and cough, can lead to a renewed decrease in HRQoL after LTx [13, 14, 21]. The GR-Scale was able to detect this decrease in HRQoL both at baseline and at follow-up, with statistically significant higher values in the group with CLAD (group 4) than without CLAD (group 3), indicating a more severely impaired HRQoL. This observation suggests that the GR-Scale may be applicable for sequential assessments of changes in HRQoL.

The symptoms “shortness of breath” and “fatigue” are typical symptoms of CLAD [21]. It was, therefore, anticipated that the GR-Scale would be able to identify differences here. This has been achieved consistently over repeated measurements. The item “overall sense of wellbeing” was statistically significant higher (worse) in the CLAD group at follow-up, whereas only numerically higher values were found at baseline. This suggests that there is a difference between the groups, which was not as pronounced at baseline as it was at follow-up. One possible explanation is that 2 patients progressed from CLAD stage 1 to stage 2 between the interviews, which likely contributed to an increase in their symptoms and could already result in a statistically significant change in a small group of 11 patients.

Cough is another typical symptom of CLAD where we found no statistically significant difference between the two groups. At follow-up, however, numerically higher values in the CLAD group were found, possibly driven by two patients who progressed from CLAD stage 1 to 2 between the interviews, which coincided with an increase in symptoms. The limited number of patients included do not allow more detailed analyses. Therefore, larger studies with more homogeneous populations regarding CLAD stage and phenotype are necessary to investigate the item “cough” in more detail.

HRQoL has become increasingly important in recent years and has been shown to be an important adjunct to routine measurements, some of which do not always correspond with the patients’ perceptions [2, 13, 14, 22–24]. Improving HRQoL is a main goal of LTx [11–13] and should be recorded regularly in lung transplant recipients to assess how patients “feel and function” in their daily lives after LTx. In our study all patients completed the questionnaire, unsupported by a health care professional, in a complete and evaluable way, same as in our previous study on the applicability of the questionnaire in different ILD entities [26]. Thus, in our hands the questionnaire is easy to understand and to apply and could be a simple and quick tool for everyday clinical practice. The GR-Scale could also be used in patients after LTx as both a screening tool to identify patients who need further evaluation and a longitudinal tool to track the patient’s health status over time [25, 26]. When applied longitudinally, it could offer a consistent assessment of the patient’s HRQoL and potentially improve monitoring of disease dynamics, including the onset and progression of CLAD in lung transplanted patients. Based on our current data, no conclusions can be made regarding the utility of the GR-Scale for the early detection of CLAD. Nevertheless, applying the GR-Scale in this context might be an interesting approach for future investigations and further studies are required to determine whether longitudinal GR-Scale assessments could contribute to early recognition of CLAD.

Our study has several limitations. First, the study populations were relatively small, enrolled in a single center, and the interval between the two interviews varied among patients, ranging from 3 to 6 months. Since the study was performed at a tertiary hospital and a transplant center, the inclusion of more complex cases or patients who received more intensive follow-up may have generated a selection bias in the ILD cohort. Second, there is no validation of the questionnaire in patients after LTx. This is an important aspect to consider and to address in further studies. In addition, we included all CLAD phenotypes and stages. Furthermore, between the groups with and without CLAD there was a relatively large variability in size as well as in the time since transplantation, potentially impacting our results. Lastly, when comparing HRQoL before and after LTx, we analyzed two different populations rather than following a single cohort longitudinally. As a result, we were unable to measure individual changes in HRQoL over time and could only assess differences between groups. A similar limitation applies to our analysis of the impact of CLAD, where we did not assess the same patients before and after the occurrence of CLAD but instead compared two separate groups.

## Conclusion

HRQoL is an essential aspect in the care for non-transplanted patients with ILD as well as for transplanted patients and it should be recorded regularly in clinical practice. In our pilot study, we used the GR-Scale for the first time in patients who had undergone LTx due to ILD and in a matched group of non-transplanted ILD patients. We were able to determine statistically significant higher values (worse condition) in the GR-Scale in the non-transplanted compared to the transplanted patients, as well as higher values in transplanted patients with CLAD than without CLAD. In conclusion, our data suggests that the GR-Scale is an easy-to-use and valuable tool to assess HRQoL in lung transplant recipients providing important additional information for clinical evaluation of this patient population, also longitudinally.

## Data Availability

The data will be made available on request and with consideration of further evaluations of our study team (contact: Direktion.Med5@med.uni-muenchen.de).

## Abbreviations

BOS: Bronchiolitis obliterans syndrome
cHP: Chronic hypersensitivity pneumonitis
CI: Confidence interval
CLAD: Chronic lung allograft dysfunction
EQ-5D-5L: EuroQol Five-Dimensional Five-Level questionnaire
ES: Effect size
FEV1: Forced expiratory volume in 1 s
FVC: Forced vital capacity
GR-Scale: German version of the R-Scale
HRQoL: Health related quality of life
ILD: Interstitial lung disease
IPF: Idiopathic pulmonary fibrosis
K-BILD: King’s Brief Interstitial Lung Disease questionnaire
LTx: Lung transplantation
MDD: Multidisciplinary discussion
NSIP: Non-specific interstitial pneumonia
PROM: Patient reported outcome measures
RAS: Restrictive allograft syndrome
R-Scale-PF: Raghu scale for pulmonary fibrosis
SD: Standard deviation
TLC: Total lung capacity
uILD: Unclassifiable interstitial lung disease

## References

[1] Swigris JJ, Stewart AL, Gould MK, et al. Patients’ perspectives on how idiopathic pulmonary fibrosis affects the quality of their lives. Health Qual Life Outcomes. 2005;3:61. 16212668 10.1186/1477-7525-3-61 PMC1276807

[2] Swigris JJ, Brown KK, Abdulqawi R, et al. Patients’ perceptions and patient-reported outcomes in progressive-fibrosing interstitial lung diseases. Eur Respir Rev. 2018;27:180075. 30578334 10.1183/16000617.0075-2018 PMC9489034

[3] De Vries J, Drent M. Quality of life and health status in interstitial lung diseases. Curr Opin Pulm Med. 2006;12:354–8. 16926651 10.1097/01.mcp.0000239553.93443.d8

[4] Szentes BL, Kreuter M, Bahmer T, et al. Quality of life assessment in interstitial lung diseases:a comparison of the disease-specific K-BILD with the generic EQ-5D-5L. Respir Res. 2018;19:101. 29801506 10.1186/s12931-018-0808-x PMC5970441

[5] Behr J. Interstitielle lungenerkrankungen: was muss der Hausarzt wissen? MMW Fortschr Med. 2018;160:38–42. 29464621 10.1007/s15006-018-0198-5 PMC7101588

[6] Behr J, Günther A, Bonella F, et al. S2K-Leitlinie Zur diagnostik der idiopathischen lungenfibrose. Pneumologie. 2020;74:263–93. 32227328 10.1055/a-1120-3531

[7] Kreuter M, Ladner UM, Costabel U, et al. The diagnosis and treatment of pulmonary fibrosis. Dtsch Arztebl Int. 2021;118:152–62. 33531115 10.3238/arztebl.m2021.0018 PMC8212400

[8] Kapnadak SG, Raghu G. Lung transplantation for interstitial lung disease. Eur Respir Rev. 2021;30:210017. 34348979 10.1183/16000617.0017-2021 PMC9488499

[9] Raghu G, Collard HR, Egan JJ, et al. An official ATS/ERS/JRS/ALAT statement: idiopathic pulmonary fibrosis: Evidence-based guidelines for diagnosis and management. Am J Respir Crit Care Med. 2011;183:788–824. 21471066 10.1164/rccm.2009-040GL PMC5450933

[10] Prasse A. Die Idiopathische Lungenfibrose Pneumologie. 2015;69:608–15. 26444136 10.1055/s-0034-1393036

[11] Jaksch P, Hoetzenecker K, Lungentransplantation. Pneumologe. 2020;17:285–96.

[12] Singer JP, Soong A, Chen J, et al. Development and preliminary validation of the lung transplant quality of life (LT-QOL) survey. Am J Respir Crit Care Med. 2019;199:1008–19. 30303408 10.1164/rccm.201806-1198OC PMC6467318

[13] Takahashi R, Takahashi T, Okada Y, et al. Factors associated with quality of life in patients receiving lung transplantation: a cross-sectional study. BMC Pulm Med. 2023;23:225. 37353819 10.1186/s12890-023-02526-0 PMC10288771

[14] Zhu X, Liang Y, Zhou H et al. Changes in Health-Related Quality of Life During the First Year in Lung Transplant Recipients. Transplantation Proceedings. 2021;53:276–87. 32768289 10.1016/j.transproceed.2020.06.037

[15] Verleden GM, Glanville AR, Lease ED, et al. Chronic lung allograft dysfunction: Definition, diagnostic criteria, and approaches to treatment―A consensus report from the pulmonary Council of the ISHLT. J Heart Lung Transplantation. 2019;38:493–503. 10.1016/j.healun.2019.03.009 30962148

[16] Diel R, Simon S, Gottlieb J. Chronic lung allograft dysfunction is associated with significant disability after lung Transplantation—A burden of disease analysis in 1025 cases. Adv Respir Med. 2023;91:432–44. 37887076 10.3390/arm91050033 PMC10603923

[17] Kotecha S, Paraskeva MA, Levin K, et al. An update on chronic lung allograft dysfunction. Ann Transl Med. 2020;8:417. 32355861 10.21037/atm.2020.01.05 PMC7186740

[18] Chambers DC, Cherikh WS, Goldfarb SB, et al. The international thoracic organ transplant registry of the international society for heart and lung transplantation: Thirty-fifth adult lung and heart-lung transplant report-2018; focus theme: multiorgan transplantation. J Heart Lung Transpl. 2018;37:1169–83. 10.1016/j.healun.2018.07.020 30293613

[19] Fu A, Vasileva A, Hanafi N, et al. Characterization of chronic lung allograft dysfunction phenotypes using spectral and intrabreath oscillometry. Front Physiol. 2022;13:980942. 36277208 10.3389/fphys.2022.980942 PMC9582781

[20] Hofstetter E, Boerner G. Survival after lung transplantation in Europe - Clad as major cause of death. J Heart Lung Transplantation. 2022;41:S286.

[21] Bedair B, Tague LK. Chronic rejection (Chronic lung allograft Dysfunction-CLAD) following lung transplant. Am J Respir Crit Care Med. 2024;209:P3–4.

[22] The WHOQOL Group. The World Health Organization Quality of Life assessment (WHOQOL): position paper from the World Health Organization. Soc Sci Med. 1995;41(10):1403–9. 10.1016/0277-9536(95)00112-k 8560308

[23] Swigris JJ, Fairclough D. Patient-Reported outcomes in idiopathic pulmonary fibrosis research. Chest. 2012;142:291–7. 22871754 10.1378/chest.11-2602 PMC3425334

[24] Raghu G, Ghazipura M, Fleming TR et al. Meaningful Endpoints for Idiopathic Pulmonary Fibrosis (IPF) Clinical Trials: Emphasis on ‘Feels, Functions, Survives’. Report of a Collaborative Discussion in a Symposium with Direct Engagement from Representatives of Patients, Investigators, the National Institutes of Health, a Patient Advocacy Organization, and a Regulatory Agency. Am J Respir Crit Care Med. 2024;209:647–69. 38174955 10.1164/rccm.202312-2213SO PMC12039048

[25] Scallan C, Strand L, Hayes J, et al. R-scale for pulmonary fibrosis: a simple, visual tool for the assessment of health-related quality of life. Eur Respir J. 2022;59:2100917. 34112729 10.1183/13993003.00917-2021

[26] Stoltefuß S, Leuschner G, Milger K, et al. Assessing health-related quality of life in patients with interstitial lung diseases. BMC Pulm Med. 2024;24:452. 39272068 10.1186/s12890-024-03262-9 PMC11401309

[27] Travis WD, Costabel U, Hansell DM, et al. An official American thoracic society/European respiratory society statement: update of the international multidisciplinary classification of the idiopathic interstitial pneumonias. Am J Respir Crit Care Med. 2013;188:733–48. 24032382 10.1164/rccm.201308-1483ST PMC5803655

[28] Cohen J. Statistical power analysis for the behavioral sciences. 2nd ed. New York, NY: Psychology Press; 2009.

[29] Finlen Copeland CA, Vock DM, Pieper K, et al. Impact of lung transplantation on recipient quality of life. Chest. 2013;143:744–50. 23188377 10.1378/chest.12-0971 PMC3747721

[30] Lanuza DM, Lefaiver C, Mc Cabe M, et al. Prospective study of functional status and quality of life before and after lung transplantation. Chest. 2000;118:115–22. 10893368 10.1378/chest.118.1.115

[31] Seiler A, Klaghofer R, Ture M, et al. A systematic review of health-related quality of life and psychological outcomes after lung transplantation. J Heart Lung Transpl. 2016;35:195–202. 10.1016/j.healun.2015.07.003 26403492

